# Genotyping and Whole-Genome Resequencing of Welsh Sheep Breeds Reveal Candidate Genes and Variants for Adaptation to Local Environment and Socioeconomic Traits

**DOI:** 10.3389/fgene.2021.612492

**Published:** 2021-06-18

**Authors:** James Sweet-Jones, Vasileios Panagiotis Lenis, Andrey A. Yurchenko, Nikolay S. Yudin, Martin Swain, Denis M. Larkin

**Affiliations:** ^1^Royal Veterinary College, University of London, London, United Kingdom; ^2^Institute of Biological, Environmental and Rural Sciences, University of Aberystwyth, Aberystwyth, United Kingdom; ^3^School of Health and Life Sciences, Teesside University, Middlesbrough, United Kingdom; ^4^The Federal Research Center Institute of Cytology and Genetics, The Siberian Branch of the Russian Academy of Sciences (ICG SB RAS), Novosibirsk, Russia

**Keywords:** sheep, signatures of selection, Wales, whole-genome resequencing, adaptation

## Abstract

**Background:**

Advances in genetic tools applied to livestock breeding has prompted research into the previously neglected breeds adapted to harsh local environments. One such group is the Welsh mountain sheep breeds, which can be farmed at altitudes of 300 m above sea level but are considered to have a low productive value because of their poor wool quality and small carcass size. This is contrary to the lowland breeds which are more suited to wool and meat production qualities, but do not fare well on upland pasture. Herein, medium-density genotyping data from 317 individuals representing 15 Welsh sheep breeds were used alongside the whole-genome resequencing data of 14 breeds from the same set to scan for the signatures of selection and candidate genetic variants using haplotype- and SNP-based approaches.

**Results:**

Haplotype-based selection scan performed on the genotyping data pointed to a strong selection in the regions of *GBA3*, *PPARGC1A*, *APOB*, and *PPP1R16B* genes in the upland breeds, and *RNF24*, *PANK2*, and *MUC15* in the lowland breeds. SNP-based selection scan performed on the resequencing data pointed to the missense mutations under putative selection relating to a local adaptation in the upland breeds with functions such as angiogenesis (*VASH1*), anti-oxidation (*RWDD1*), cell stress (*HSPA5*), membrane transport (*ABCA13* and *SLC22A7*), and insulin signaling (*PTPN1* and *GIGFY1*). By contrast, genes containing candidate missense mutations in the lowland breeds are related to cell cycle (*CDK5RAP2*), cell adhesion (*CDHR3*), and coat color (*MC1R*).

**Conclusion:**

We found new variants in genes with potentially functional consequences to the adaptation of local sheep to their environments in Wales. Knowledge of these variations is important for improving the adaptative qualities of UK and world sheep breeds through a marker-assisted selection.

## Introduction

Since the domestication in the semi-arid Fertile Crescent of Iran and Turkey, sheep (*Ovis aries*) have undergone migration and selection to form established breeds that are well-suited to various local environments (Zeder, 2008). The process of natural or artificial positive/negative selection results in genomic regions of a decreased diversity, which are known as the signatures of selection (Kijas et al., 2012). Detecting signatures of selection is important for understanding the genetic mechanisms of the adaptation of breeds to their local environments. Previous investigations have unearthed genes relating to hypoxia in sheep adapted to high altitudes on the Qinghai-Tibetan Plateau of China (*THRB*) (Yang et al., 2016), fat deposition in sheep from arid deserts of northern Africa (*PCDH9*) (Kim et al., 2016), and metabolism in Russian sheep adapted to low temperatures (*POMC*) (Yurchenko et al., 2019). Whilst these examples represent the extreme circumstances, there are examples of selection in sheep adapted to more temperate climates. For instance, three upland sheep breeds from northern England were shown to demonstrate higher than expected frequencies of the known missense mutations in genes associated with reproductive success (*PRLP*) and presence of horns (*RXFP2*) (Bowles et al., 2014). Knowledge of such selection is essential for the continued improvement of breeds better suited to their environments and with a better socioeconomic trait potential in current selection programs, which is particularly pressing in terms of climate change (Bowles, 2015). Here, we investigated another example of previously neglected breeds adapted to local environments in Wales, United Kingdom, where sheep have historically been farmed at altitudes of >1,000 feet (∼300 m) above sea level on a rough pasture with shallow-rooted plants (Davies, 1935).

Sheep were introduced to Wales by Neolithic settlers, bringing a primitive breed similar to the contemporary Soay (6,000 years ago) with two likely further introductions by Roman white-faced, fine-wool breeds (2,000 years ago) and Norse black-faced breeds (1,000 years ago) (Ryder, 1964). The South Wales Welsh Mountain (SWWM) breed of today, a descendent of the Roman and Soay breeds, has been documented since the 16th century and is renowned for its hardiness, lambing ability, and sweet meat taste. Despite this potential economic gain, these mountain sheep suffer a trade-off to a kemp wool and small carcass size, leading them to be poorly exploited outside of Wales (Williams-Davies, 1981). To overcome this, the SWWM breed was cross-bred in the eighteenth century to form a number of more productive local breeds, which are now farmed on the lowlands (Ryder, 1964; Williams-Davies, 1981).

Previous work to reveal the population history of Welsh sheep showed that Welsh breeds clustered closely with each other based on haplotype sharing, forming two groups within this cluster, which aligned with the upland or lowland farming style. Exceptions to this were the Black Welsh Mountain (BWM) and Kerry Hill with Beulah, which remained distinct from all other breeds (Beynon et al., 2015). This distinction of BWM is supportive of the alternate ancestry from Norse breeds and deliberate selection based on coat color (Williams-Davies, 1981). Moreover, the divergence of Kerry Hill and Beulah may be the result of a genetic bottleneck or a founder effect that these breeds underwent. Additional support of this can be seen in the low effective population sizes and high inbreeding coefficients of these breeds (Beynon et al., 2015). Low effective population sizes endanger these breeds to risks of increased homozygosity and lack of genetic diversity due to inbreeding. This is a potential risk for UK upland sheep breeds who have remained geographically and genetically isolated due to their adaptability to thrive on pastures that other breeds cannot (Bowles et al., 2014; Heaton et al., 2014).

With this in mind, it is important, from a perspective of cultural significance, breed conservation, and breed improvement, to study the adaptation of Welsh breeds, which potentially offers insight into genomic adaption to upland farming, as well as lambing and meat quality. Likewise, lowland productive breeds offer a good comparison due to their stronger capabilities for traits related to socioeconomic gain. Through the use of a commercially available medium-density SNP genotyping array with a HapFLK and Decorrelated Composite of Multiple Statistics (DCMS) software and whole-genome resequencing data with a DCMS pipeline, this paper aims to identify the signatures of selection in the genomes of Welsh sheep breeds and candidate genes containing functional missense mutations in these regions.

## Materials and Methods

### Data Source and Variant Calling

Genotyping data from 353 individuals across 18 Welsh breeds on the Illumina OvineSNP50 SNP array from Beynon et al. (2015) were used in this study. Illumina pair-ended read (150 bp) resequencing of 11 Welsh sheep samples from the same set, representing one sample per breed (Supplementary Table 1), was performed at the University of Aberystwyth to ∼13× raw coverage using Illumina HiSeq according to the manufacturer’s protocol. Three remaining Welsh sheep resequenced genomes for Welsh Hardy-Speckled Faced, Dolgellau Welsh Mountain, and Talybont Welsh Mountain were downloaded from the National Centre for Biotechnology Information Sequence Read Archive, PRJNA160933 (Heaton et al., 2014). A dataset of resequenced Russian sheep samples (*n* = 40) adapted to a contrasting environment was used as an outgroup in this study (Sweet-Jones et al., 2021).

Reads were mapped by using the Burrows-Wheeler Aligner BWA-MEM (BWA V.0.7.10 (Li, 2013) to the reference sheep genome Oar_V.3.1. Reads were sorted using the Samtools V.0.1.18 (Li et al., 2009) and duplicates were marked with the Picard V.2.18^1^. Libraries were also merged using Picard. Base Quality Score Recalibration (BQSR) was performed, which account for the systematic errors in sequencing, using the Genome Analysis Toolkit (GATK V.3.8; McKenna et al., 2010). Samples then underwent variant calling with the GATK HaplotypeCaller function. Finally, all samples (*n* = 54) underwent joint calling to merge all reported variants into a single vcf file. Following this, hard filtering for quality scores assigned by BQSR was performed by GATK, using the filter expression “[QD < 2.0| | FS > 60.00| | MQ < 40.00| | MQRankSum < −12.5| | ReadPosRankSum < −8.0].” All variants from resequenced data were converted into a Plink V.1.90 (Purcell et al., 2007) format to be run through the DCMS pipeline (Yurchenko et al., 2019).

### HapFLK Statistics

Welsh breed genotyping data were separated into three groups of related breeds based on the clustering analysis performed by Beynon et al. (2015), resulting in one group of upland breeds and two groups of lowland breeds. Of the two lowland groups, one consisted of five lowland breeds and the other consisted of only the Kerry Hill and Beulah breeds (KHB) (Table 1). Plink-formatted (Purcell et al., 2007) files for the upland and lowland breed groups had genotypes from the sex chromosomes removed and were filtered to remove the rare alleles [–maf 0.05], low called SNPs [–geno 0.01], or poorly genotyped individuals [–mind 0.05]. FastPhase V.1.4 (Scheet and Stephens, 2006) was used to estimate the number of haplotype clusters (k) for each group (Lowland k = 48, KHB k = 25, and Upland k = 53). HapFLK software (Bonhomme et al., 2010) was used to obtain selection statistics for each group. This test uses the hierarchal population structure to identify the haplotype-based selective sweeps, which focuses on the inherited combinations of alleles. It has the advantage of an increased statistical power, reliably detecting the hard and soft selective sweeps, and is a realistic simulation of selection through the haplotypes. HapFLK *p*-values were calculated using the Python script scaling_chi2_hapflk.py (Bonhomme et al., 2010; Fariello et al., 2013). Adjusted *p*-values, or *q*-values, were calculated through the R qqman *q*-value function (Turner, 2014). Selected intervals were determined by boundaries of *q* < 0.05 with SNPs within an interval of *q* < 0.01 considered to be under a strong selection.

**TABLE 1:** Breed representation in the genotyping and resequencing datasets.

Breed	Abbreviation	Genotyped samples	Horns	Base color	Fleece	HapFLK group	Resequencing group
Badger Faced Welsh Mountain	BFWM	21	Yes	Black and white	Firm	Upland	NA*
Balwen	−	14	No	Black and white	Firm	Upland	Upland
Beulah	–	22	No	Black and white	Fine	KHB	Lowland
Black Welsh Mountain	BWM	24	Yes	Black	Firm	Upland	Upland
Brecknock Hill Cheviot	BHC	24	No	White	Fine	Upland	Upland
Clun Forest	−	17	No	Black	Fine	Lowland	Lowland
Dolgellau Welsh Mountain	DWM	−	Yes	White	Firm	NA*	Upland
Hardy Speckled Faced	HSF	24	Yes	White	Fine	Lowland	Lowland
Hill Radnor	−	21	No	Gray-brown	Fine	Lowland	Lowland
Kerry Hill	−	18	No	White	Fine	KHB	Lowland
Llandovery White Faced	LWF	24	No	White	Fine	Upland	Upland
Llanwenog	−	21	No	Black	Fine	Lowland	Lowland
Lleyn	−	22	No	White	Fine	Lowland	Lowland
South Wales Welsh Mountain	SWWM	17	Yes	White	Firm	Upland	Upland
Talybont Welsh Mountain	TWM	24	No	White	Firm	Upland	Upland
Welsh Mountain Hill Flock	WMHF	24	Yes	White	Firm	Upland	NA
Average/Total	−	21/317	No	−	−	102/40/173	7/7

*NA denotes the exclusion of breed from either the HapFLK or resequencing study due to the unavailability of samples.

### De-correlated Composite of Multiple Signals (DCMS)

Five established measures of selection and genetic diversity were both used on the genotyping (15 breeds) and whole genome resequencing (upland breeds *n* = 7; lowland breeds = 7) datasets as a DCMS (Table 1; Ma et al., 2015). Statistics used were: (i/ii) *H2*/*H1* and *H12*, which can distinguish the hard and soft selective sweeps by measuring the intensity of selection (Garud et al., 2015); (iii) Tajima’s *D* comparing pairwise sequence differences and the number of segregating sites, detecting positive, negative, or balancing selection (Tajima, 1989); (iv) nucleotide diversity (*Pi*), average number of nucleotide differences between two sequences (Nei and Li, 1979); (v) *F*_*ST*_ fixation index, comparing single SNP frequencies across a population (Weir and Cockerham, 1984). By weighting the result of each statistic and generating a combined score, regions that overlap in the analysis outcomes gain a stronger evidence to be a region under selection.

### H2/H1 and H12

Autosomal SNPs were filtered for –maf 0.0000001 –geno 0.1 –mind 0.1 by Plink. SNPs were then phased by chromosome using ShapeIt2 (Delaneau et al., 2011) with 400 states and an effective population size of 100. Phased chromosomes were split into appropriate groupings per chromosome using Plink and H2/H1, H12 were calculated using the H1_H12.py Python script (Garud et al., 2015). H2/H1 and H12 values were calculated in windows of 25 SNPs using a step size of one SNP following our previous study (Yurchenko et al., 2019).

### Tajima’s *D*

Tajima’s *D* for mutation index was calculated over the same intervals of 25 SNPs, whose lengths were calculated from the output of the H2/H1 and H12 statistics. Using the vcftools V.0.1.13 (Danecek et al., 2011), Tajima’s D was calculated [–TajimaD 900000000] for each chromosome per group/breed file per window. Output files were concatenated per group.

### Fixation Index

*F*_*ST*_ was calculated with Plink comparing each group to all others. All negative values were converted to zero, and data were smoothed with the R *runmed* function in windows of 31 SNPs.

### Nucleotide Diversity

Plink-format file was split by chromosome per breed, and nucleotide diversity was calculated with the vcftools [–site-pi] option. Data were then smoothed using the R *runmed* function in windows of 31 SNPs.

### Combining Statistics With DCMS

Output files from individual statistics were sorted and joined by SNP id. Genome-wide *p*-values through ranking results of each statistic were calculated in the R MINOTAUR *stat-to-p*-value function specifying the one-tailed tests (H2/H1, H12, *F*_*ST*_–right tailed, Pi, and Tajima’s D–left tailed). Covariance matrix was constructed based on sampling 300,000 randomly sampled SNPs using the R *CovNAMcd* function where the alpha = 0.75. DCMS statistics were calculated using the *DCMS* function and fitted to a normal distribution to examine normality implemented by the R MASS *rlm* function. These fitted DCMS values were converted to *p*-values using *pnorm*, and adjusted for a false discovery rate with the *qvalue* function. *Q*-values were parsed to determine selection with region boundaries set at a *q* < 0.2 and a threshold of *q* < 0.01.

### Candidate Gene Search

For the regions defined by our pipelines as being under selection, genes were identified using a list of 26,958 genes of the Oar_V.3.1 genome downloaded from Ensembl BioMart v.98. Within each selected region, genes were then ranked based on their distances to the most significant SNP of that region with the closest gene being the top-ranking. The top 10 highest ranking genes from each region underwent literature review for their previous associations to adaptation to local environments or socioeconomic traits in animals. Genes with established links to these traits were identified as candidate genes in this study.

In the resequencing study, all SNPs were annotated with NGS-SNP (Grant et al., 2011) to identify missense mutations. Only genes with missense mutations in the regions under selection were considered for literature review. Additionally, missense SNPs with a strong support from the *F*_*ST*_ statistic (*F*_*ST*_ ≥ 0.3) were analyzed with PolyPhen2 to predict their effects on protein structure and function (0 = benign and 1 = deleterious; Adzhubei et al., 2013).

Functional enrichment analysis was performed using the Database for Annotation, Visualization, and Integrated Discovery (DAVID) v. 6.8 (Huang et al., 2009) using the same gene list downloaded from Ensembl Biomart v. 98. Enrichments were detected using the DAVID functional clustering tool, which verifies enrichments of similar GO terms across many different databases, to confer a stronger evidence of enrichment. Scores > 1.3-fold enrichment were considered.

### Copy Number Variant (CNV) Analysis of Resequenced Genomes

CNV detection was performed to identify the regions in the genomes of Welsh sheep that had been duplicated or deleted with respect to the Texel reference genome. We identified CNVs in the resequenced samples with the cn.Mops R package (Klambauer et al., 2012) in a window length of 700 SNPs, using sequences that had undergone BQSR. This resulted in each individual being given a raw copy number (CN) per window. CN1–CN3 were considered to be normal and discounted from the results. Raw CNVs were merged into the CNV Regions (CNVRs) with the BedOps *bedmap* function using at least 50% reciprocal overlap in at least three individuals within the same group as criteria for inclusion. Duplicate CNVRs were removed, and the neighboring CNVRs were merged. This allowed us to be confident in our results by excluding the regions where CNVRs appear to overlap the signatures of selection, as these cast doubt over their reliability.

### Data Visualization

HapFLK clusterplots and haplotype trees were visualized from the prepared R scripts available (Bonhomme et al., 2010; Fariello et al., 2013). All Manhattan plots were rendered in R by the *qqman* package setting the suggestive line (*q* = 0.05) and genome-wide line (*q* = 0.01) to indicate selection. Haplostrips for visualization of haplotype sharing (Marnetto and Huerta-Sánchez, 2017) in the regions under selection detected by DCMS were run using phased data.

## Results

### Regions Under Selection Detected From Genotyping Data

**FIGURE 1:**
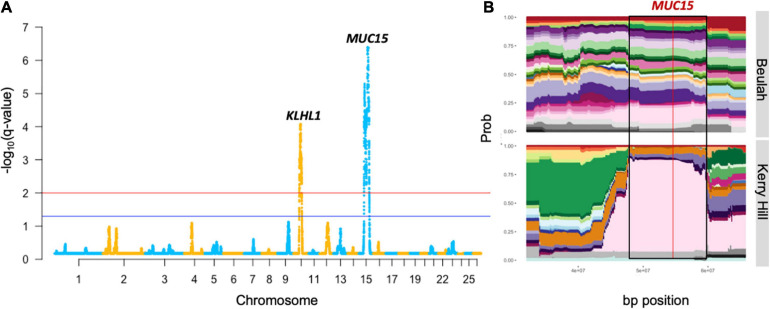
Output of genomic regions under strong selection in the KHB group from the HapFLK study. This includes the Manhattan plot **(A)** with a gene proximal to the most significant SNP of the highly selected peaks. Cluster plot **(B)** shows the tracks of haplotype diversity for the peak highlighted on the Manhattan plot by black borders where the red line represents the most significant SNP.

**FIGURE 2:**
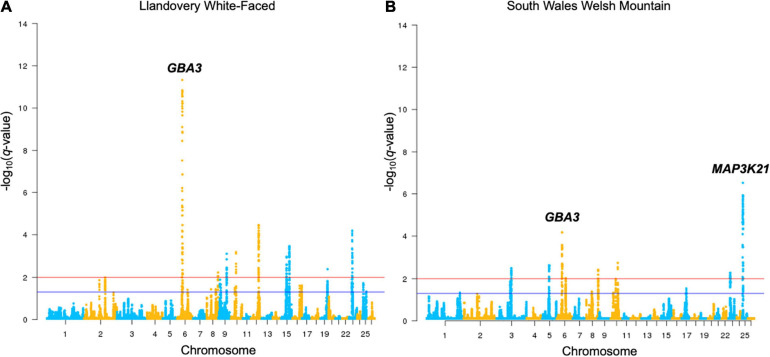
Manhattan plots of Llandovery White-faced **(A)** and South Wales Welsh Mountain **(B)** genotyped breeds’ DCMS output. All other breeds are shown in Supplementary Figure 2. Selection thresholds for a suggestive (*q* < 0.05) and strong (*q* < 0.01) selection shown by blue and red lines, respectively. Significant selection peaks have been annotated with the name of the top-ranked gene of that region.

Using the HapFLK software, signatures of selection were found in each grouping using 44,711 SNPs (lowland group contained one region, KHB-two, and upland-31; Supplementary Tables 2–4). Cluster plots and Haplotype Trees for the most significantly selected regions in the KBH group are shown in Figure 1 and for other groups, in Supplementary Figure 1. Lengths of the regions found under selection ranged from 1.81 to 64.78 Mb with a median length of 7.33 Mb. Moreover, DCMS also found the regions under selection in all 15 breeds, with at least one region of a strong selection (*q* < 0.01) in each breed using 47,366 SNPs. Lengths of the regions under selection ranged from 1 to 3.9 Mb where the median length of regions was 0.16 Mb. The number of regions detected in each breed ranged from 8 in Kerry Hill to 89 in Clun Forest (Supplementary Tables 5–19). In these regions under selection, 1,089 unique genes were found, with 179 occurring in multiple breeds including: *GBA3* (Hill Radnor, LWF, Lleyn, SWWM, TWM, and WMHF), *ENSOARG00000021104* (BFWM, Clun Forest, Hill Radnor, and Lleyn), *PCDH9* (BHC, Hill Radnor, and Llanwenog), and *PPARGCA1* (LWF, SWWM, and WMHF). Manhattan plots for all the breeds investigated are shown in Figure 2 and Supplementary Figure 2.

There was a substantial overlap between the results for a region found on OAR6: 31.99–46.00 Mb (*q* = 2.0 × 10^–5^) in the upland breeds in the HapFLK study to a narrower region, OAR6: 40.20–43.38 Mb, in LWF (*q* = 6.9 × 10^–15^), WMHF (*q* = 4.3 × 10^–11^), and SWWM (*q* = 8.73 × 10^–5^). In all cases, the top ranked genes were *GBA3* linked to liver metabolism (Dekker et al., 2011) and *PPARGC1A* with known roles in mitochondrial biogenesis, fat deposition, and milk fatty acid composition in cattle (Fernandez-Marcos and Auwerx, 2011; Armstrong et al., 2018; Yuan et al., 2019; Li et al., 2016). On the HapFLK cluster plot for this region, low diversity was seen in the Balwen, BWM, LWF, and SWWM breeds, further supporting the idea of selection for this region. When plotted on a haplotype tree, it can be seen that Balwen had the longest branch compared to the other upland breeds, whilst the strongest signature of selection is seen in SWWM (Supplementary Figure 1).

Several other candidate regions detected by HapFLK overlapped with the regions found in the Welsh breeds by DCMS. OAR3: 23.23–33.86 Mb (*q* = 5.0 × 10^–6^) shared its top-ranked genes, *TDRD15* and *APOB*, with a region under selection in the upland Balwen breed (*q* = 0.0003). These genes are associated with cholesterol mobilization in Large White pigs (Bovo et al., 2019). OAR7: 47.55–54.20 Mb (*q* = 0.0008) overlapped with another region OAR7: 51.30–52.76 Mb in the upland BWM (*q* = 5.0 × 10^–9^), but these did not share the top-ranked genes. Strong overlapping candidate genes are presented in Table 2.

**TABLE 2:** Candidate genomic regions and genes from the HapFLK analysis and corresponding DCMS region.

OAR	Region start	Region end	Group	*q*-value	Candidate genes (rank)	Function	Overlapping DCMS result
1	258,588,662	275,499,089	Upland	0.0002	*SIM2(1), CLDN14(2)*		
3	23,229,012	33,863,536	Upland	4.0 × 10^–6^	*TDRD15(1), APOB(2)*	Cholesterol efflux	Balwen
6	31,991,037	45,997,407	Upland	1.0 × 10^–5^	*GBA3(1), PPARGC1A(2), DHX15(3), SOD3(4), KCNIP4(6)*	Metabolism	LWF, WMHF, SWWM
10	35,062,152	51,872,705	KHB	8.0 × 10^–5^	*KLHL1(1), PCDH9(2)*	Neurodevelopment, cell adhesion	
13	46,620,758	72,941,741	Upland	2.0 × 10^–6^	*DHX35(1), PPP1R16B(3), ADIG(6)*	Innate immunity, angiogenesis	BFWM
13	47,164,400	54,366,328	Lowland	0.01	*RNF24(1), PANK2(2), MAVS(3)*	Vision	HSF
15	32,152,675	65,710,885	Upland	4.0 × 10^–7^	*MUC15(1), ANO3(3)*	Mucous production	

The most strongly selected region in the HapFLK study was seen on OAR15 32.15–72.10 Mb in the KHB lowland group, with the top-ranking gene being *MUC15* (*q* = 4.1 × 10^–7^), associated with a low fecal egg count in Spanish Churra sheep during gastrointestinal parasite infections (Periasamy et al., 2014; Benavides et al., 2015). This was supported by a low haplotype diversity seen in the Kerry Hill breed at this locus, but this signature was not seen in Beulah or in the DCMS results in either breeds.

In the other lowland group, the only region found under selection, OAR13: 47.16–54.37 Mb (*q* = 0.01), overlapped with another region found in the upland group, OAR13: 46.62–72.94 Mb (*q* = 2.0 × 10^–6^), but the top candidate genes were different. In the lowland breeds, the top-ranked genes were *RNF24* and *PANK2*, which have been found under selection in world sheep breeds in association to a loss of vision following domestication (Naval-Sanchez et al., 2018; Wang et al., 2019). Cluster plots for this region showed a decreased haplotype diversity amongst the selected region on OAR13 in the lowland breeds, especially Clun Forest and HSF. Furthermore, significant selection at this locus in HSF (*q* = 0.0006) was confirmed using the DCMS pipeline. In the case of the upland breeds, the top-ranked genes were *DHX35* and *PPP1R16B* which are related to innate viral immunity (Rahman et al., 2017) and endothelial cell proliferation (Pszczola et al., 2018), respectively.

The genes found within the regions under selection from HapFLK, functional enrichments seen for the KHB group included the DENN domain and connexin gap junctions, enriched 1.6- and 1.4-fold, respectively. The most highly enriched terms in the upland breeds were bactericidal permeability protein, major intrinsic protein, and semaphorins, which were all enriched over threefold. The most enriched cluster seen in the genotyping DCMS analysis was the Type II keratin filaments from the Hill Radnor breed, which was enriched fivefold. This was followed by the Ribonuclease A in Clun forest, enriched 2.8-fold and leucine rich-repeats in Lleyn, enriched 2.7-fold (Supplementary Table 20).

### Signatures of Selection Detected From Resequencing Data

Fifty-four (14 Welsh and 40 Russian sheep) resequenced genomes were aligned to the Oar_V.3.1 genome with a mean filtered coverage of 11.9× (Supplementary Table 1). A total of 41,643,098 SNPs were called, which were pruned to 38,276,494 SNPs after filtering. CNVRs covered 0.27% of the lowland and 0.24% of the upland genomes, overlapping 852 and 669 genes, respectively (Supplementary Tables 21,22). Three CNVRs from the lowland breeds and 52 CNVRs from the upland breeds overlapped the regions of selection. Some of these CNVRs had a high frequency in the population, including the regions under a strong selection on OAR24 in the lowland breeds, spanning the *CLCN7* gene and on OAR17, whereas in the upland breeds, these spanned the *IGLV4-69, ZNF280B*, and *PRAME* genes.

**FIGURE 3:**
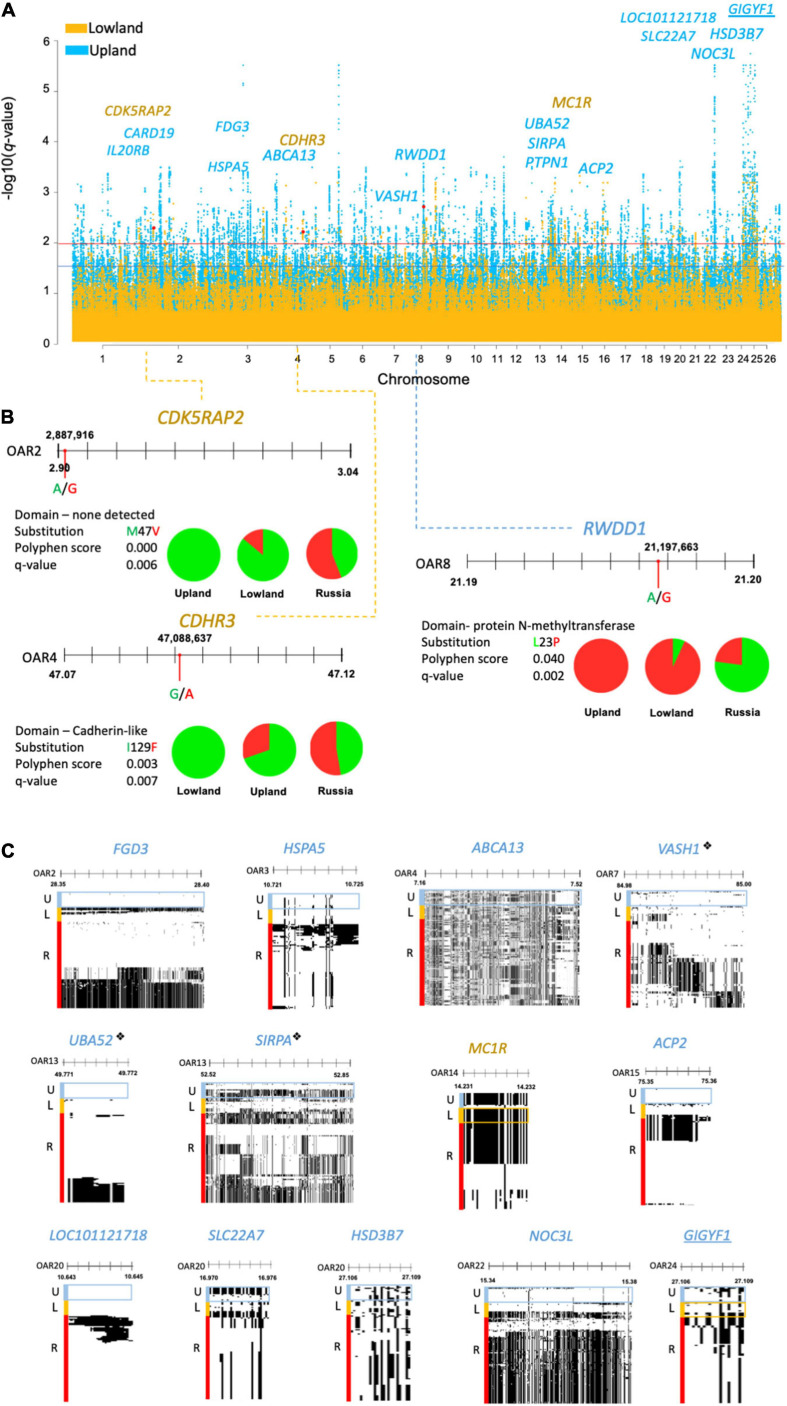
**(A)** Manhattan plot of DCMS *q*-values of the lowland (yellow) and upland (blue) breeds showing missense mutations found under selection, highlighted in red with the corresponding gene names. Selection thresholds for a suggestive (*q* < 0.05) and strong (*q* < 0.01) selection shown by blue and red lines, respectively. Underlined gene names show selection in both the lowland and upland breeds. **(B)** Allele frequencies for missense mutations identified by a strong *F*_*ST*_ score represented by pie charts (green = reference allele, red = derived allele). This shows the location of missense mutations along their gene with nucleotide substitution highlighted by a red circle. Blue and yellow dotted lines point to the corresponding peak positions of the Manhattan plot. Amino acid substitution is shown, alongside the Polyphen score and *q*-value. **(C)** Haplostrip plots spanning genes containing the missense mutations but were selected on the basis of the H2/H1 and H12 haplotype statistics. Similar haplotypes are clustered together per population to demonstrate selection within these regions across the whole gene. These show the presence of reference (white) or derived (black) alleles making up different haplotypes. Populations of interest are highlighted in boxes corresponding to their colors on the Manhattan plot. “❖” is used to denote genes under selection by DCMS and HapFLK.

After excluding the regions overlapping CNVRs, DCMS found 2,996 regions under selection in the Welsh breeds (lowland = 514, upland = 2,482; Supplementary Tables 23,24 and Figure 3A). These regions overlapped 104 and 430 genes in the lowland and upland breeds, respectively. The most significantly selected region in the upland group was OAR22: 15.3676–15.3679 Mb, which overlapped the *NOC3L* gene (*q* = 1.8 × 10^–8^), and for the lowland group, it was a single synonymous SNP located in *MYH11* (*q* = 0.0006).

### Identification of Candidate Genes and Missense Mutations

In regions under selection, there were 12 missense mutations found in the lowland breeds (Supplementary Table 25) and 85 missense mutations found in the upland breeds (Supplementary Table 26). Of these, only missense mutations and their enclosing genes, which were top-ranking SNPs in their selected intervals are discussed below, leading to a total of 4 in the lowland breeds and 14 in the upland breeds. Clarification of the type of selection relied on a strong support from the *H2/H1* and *H12* statistics, which were considered to be haplotype-based selection, or *F*_*ST*_, which was considered fixation of a variant in a population and was seen as a selection acting on individual SNPs.

Three missense mutations were found in the regions under selection (*q* < 0.01), with a strong support from the *F*_*ST*_ statistic (*F*_*ST*_ ≥ 0.3). In the lowland breeds, these included the reference alleles of OAR2:2,887,916 in the *CDK5RAP2* gene (*q* = 0.006; *F*_*ST*_ = 0.3) and OAR4:47,087,846 in *CDHR3* (*q* = 0.007; *F*_*S*__*T*_ = 0.3). For the upland breeds, only one missense mutation was found, relating to the derived allele of OAR8:21,197,663 in the *RWDD1* gene (*q* = 0.002; *F*_*ST*_ = 0.5). The Polyphen score of this selected mutation, L23P, was low and did not support the large change in the protein function (Figure 3B).

Missense SNPs in the regions under selection supported by the haplotype statistics (*H2/H1*, *H12*) were only found in the *MC1R* gene in the lowland breeds, and in the upland breeds: *FGD3*, *HSPA5*, *ABCA13*, *PTPN1*, *ACP2*, *LOC101121718*, *NOC3L*, *VASH1*, *SIRPA*, and *UBA52* genes. Alternatively, some missense SNPs received strong support from Tajima’s *D* and *Pi*, including one in the *GIGYF1* gene found in the selected regions in both the upland and lowland breeds, as well as the *SLC22A7* and *HSD3B7* genes in the upland breeds (Figure 3C).

In the upland breeds, three of the genes found with missense SNPs were also identified in the regions under selection defined in the upland breeds of the genotyping analysis. These included: *VASH1* (*q* = 0.003) on OAR7, which also overlapped with BWM from the genotyping DCMS data; *UBA52* (*q* = 0.01) on OAR13, which also overlapped with BHC from the genotyping data, and *SIRPA* (*q* = 0.007). No missense mutations found in the lowland breeds overlapped with the regions under selection detected from the genotyping dataset.

### Candidate Genes in Welsh Lowland Sheep Breeds

*MC1R*, found in a region under selection in the lowland breeds (Table 3), is known to cause an upregulation of tyrosinase in hair melanocytes, which led to an increase of black eumelanin pigment; however, in sheep, typically, white pheomelanin is produced due to selection for mutations in *MC1R* (Weatherhead and Logan, 1981; Yang et al., 2013). Another gene, *CDK5RAP2*, with a strong support from allele frequency statistics, is related to human neurodevelopment by recruiting tubulin subunits, which are important for cortical gyration (Issa et al., 2013). Mutations in this gene have also been linked to Hertwig’s anemia mutant mouse models displaying blood cytopenia, aneuploidy due to impaired cell-cycle spindle checkpoints, and increased neuronal cell death (Lizarraga et al., 2010). Finally, *CDHR3*, encoding a cell adhesion protein is linked to childhood asthma and rhinovirus-C susceptibility, was also located in a region under selection in Asian sheep by HapFLK (Fariello et al., 2014; Bochkov et al., 2015).

**TABLE 3:** Candidate genes and missense mutations in the lowland and upland sheep breeds.

OAR	Region start	Region end	SNP	Breed	Gene (rank)	Reference allele	Alternative allele	Mutation	PolyPhen	*q*-value	Function
2	2,887,916	2,887,916	2,887,916	Lowland	*CDK5RAP2*(1)	A*	G	M47V	0	0.006	Cell cycle
2	28,318,917	28,318,992		Upland	*FGD3*(1)					0.003	GTP binding
3	10,725,499	10,725,501		Upland	*HSPA5*(1)					0.007	Cell stress
4	7,476,204	7,476,211	7,476,211	Upland	*ABCA13*(1)	T*	C	K2619Q	0.9	0.006	Membrane transport
4	47,088,764	47,089,276		Lowland	*CDHR3*(1)					0.004	Cell adhesion
7	84,989,884	84,989,669		Upland	*VASH1*(1)					0.004	Angiogenesis
8	21,197,663	21,198,137	21,197,663	Upland	*RWDD1*(1)	A	G*	L23P	0.04	0.002	Anti-oxidation
13	49,771,782	49,771,855		Upland	*UBA52*(1)					0.1	Ubiquitinoylation
13	52,848,831	52,848,943		Upland	*SIRPA*(1)					0.006	Cell recognition
13	52,848,831	52,848,943		Upland	*PTPN1*(1)					0.1	Insulin pathway
14	14,231,667	14,232,187		Lowland	*MC1R*(1)					0.004	Coat color
15	75,351,718	75,352,196		Upland	*ACP2*(1)					0.006	Development
20	10,644,119	10,644,602		Upland	*LOC10110726*(1)					0.009	Fat deposition
20	16,972,103	16,972,103		Upland	*SLC22A7*(1)					0.009	Cyclic nucleotide transport
22	15,368,530	15,368,926		Upland	*NOC3L*(1)					2.0 × 10^–8^	Cell cycle
24	27,106,763	27,106,886		Upland	*HSD3B7*(1)					0.002	Bile synthesis
24	35,794,428	35,794,428		Lowland/Upland	*GIGFY*(1)					0.007	Insulin pathway

*Denotes selected allele.

### Candidate Genes in Welsh Upland Sheep Breeds

Within the upland breeds, many genes found in the selected regions had functions related to cell stress and metabolism (Table 3). The most highly supported by DCMS, *RWDD1*, encodes a transcription factor related to sulfide metabolism in metozoans (Kang et al., 2008; Li et al., 2018). *HSPA5* also responds to cell stress mechanisms by promoting protein refolding in the endoplasmic reticulum in cancers or virally-infected cells (Booth et al., 2015). This gene has also been found under selection in Chinese Yellow-Feathered chickens with regard to meat quality (Huang et al., 2020) and muscling in world pig breeds (Li et al., 2011).

Of the three genes found in the selected regions in both the genotyping and resequencing datasets, two related to cell stress mechanisms. The most significant region contains *VASH1*, encoding the vasohibin 1 signaling molecule, with known roles of negatively regulating angiogenesis (Chen et al., 2020), as well as promoting expression of antioxidation enzymes (Miyashita et al., 2012). Age-related downregulation of *VASH1* leads to a lower endothelial cell stress tolerance, posing as a risk factor for human vascular diseases in later life (Takeda et al., 2016). Secondly, *UBA52* encodes a protein with an ubiquitinate activity, with its downregulation causing cell cycle arrest and reduced protein synthesis essential for pre-implantation embryogenesis success in mouse models (Kobayashi et al., 2016).

Several genes related to metabolism and growth were found under selection in the upland breeds. The most significant of these, *HSD3B7*, is part of the *bile biosynthesis* pathway (Cheng et al., 2003). *PTPN1* is a risk-factor gene linked to diabetes and obesity (Olivier et al., 2004), which has a direct involvement in the *insulin* and *leptin signaling pathways*, and that mice lacking this gene were resistant to weight gain and intolerant to glucose (Elchebly et al., 1999). *GIGYF1* also has roles in enhancing the insulin receptor pathway, but additionally has been linked to translational repression (Giovannone et al., 2003; Tollenaere et al., 2019). Similar effects have been seen with *APC2*, which is linked to muscle mass in mice (Kärst et al., 2011). The Rho-GEF-containing gene *FGD3*, expressed in the growth plate of long bones, was previously found under selection in French Trotter and Gidran horses, as well as in Jutland and Japanese black cattle in association to birth weight (Takasuga et al., 2015; Grilz-Seger et al., 2019; Stronen et al., 2019).

Two membrane transport proteins, ABCA13 and SLC22A7, were found to be in the regions under selection in the upland breeds. *ABCA13* encodes a member of the ATP-binding cassette membrane transporter family, responsible for the active transport of biological substrates across cell membranes (Prades et al., 2002). Secondly, *SLC22A7*, a transmembrane solute carrier, with roles in cAMP and cGMP transport in mammalian tissues and, therefore, is important for intracellular signaling which may mobilize intracellular Ca^2+^, activate protein kinases, or activate transcription factors (Kobayashi et al., 2005; Yan et al., 2016). The final gene shared between the genotyping and resequencing data was *SIRPA*, which is expressed by macrophages and polarizes M1 phagocytic macrophages to M2 antiphagocytic macrophages, which is a key survival strategy for tumors (Barclay and van den Berg, 2014).

### Gene Ontology Enrichments Show Adaptations to Environment in the Resequenced Lowland and Upland Breeds

Nine functional category enrichments were found from genes within CNVRs in the lowland breeds and 14 enrichments in the upland breeds (Supplementary Tables 27,28). Some of these enrichments were shared between the lowland and upland breeds. These were semaphorins; ion transport, pleckstrin homology domain; SAND domains; and EGF-like domains. Exclusive enrichments in the lowland breeds’ CNVRs included cell surface receptors, neuromuscular process, and DNA binding whereas exclusive enrichments in the upland breeds included Src homology-3 domain Rho signal transduction, Notch signaling, and chondrocyte differentiation (1.4-fold). Genes within the regions under selection in the upland breeds showed significantly enriched clusters including interleukin-1 and ATP-binding (Supplementary Table 29). No functional category enrichments were found in genes in the regions under selection in the lowland breeds.

## Discussion

Our study has demonstrated that regions under putative selection in Welsh sheep genomes contain candidate genes for adaptation to their local environment and production of socioeconomic traits. We used a large set of animals genotyped on a relatively small number of SNPs, applying the haplotype and point-based selection scan algorithms. This was combined with a relatively small number of resequenced individuals subjected to point-based selection scan. As a result, we detected genomic regions under selection in individual and groups of breeds, including candidate missense variants within these regions. Regions under selection detected by the three approaches followed the expected patterns seen previously that the haplotype-based approach would detect larger but fewer regions than the point-based approach, which detected smaller, but more numerous regions under selection (Yurchenko et al., 2019).

Exposure to altitudes has a range of deleterious effects caused by hypoxia, exposure to ultraviolet radiation, and generation of oxygen radicals. These, in turn, have been linked to a negative energy balance, dysregulated proteostasis, cellular stress mechanisms, and DNA damage (Askew, 2002; Pasiakos et al., 2017). Therefore, the presence of genes related to cell-stress and anti-oxidation in the regions under selection gives reassurance that the results from this study are relevant to the adaptations of Welsh sheep to altitudes. Genes related to hypoxia, however, were not identified in the regions under selection in the upland breeds, so it can be assumed that it is not a stress factor for these breeds because the altitudes they are farmed at are only moderate. Furthermore, body conditioning genes, such as *FDG3* and *ACP2*, in the upland breeds also suggest physical mechanisms of adaptation, such as increased fat deposition and muscle mass, however, these could also be linked to socioeconomic performance (Giovannone et al., 2003; Bento et al., 2004; Gu et al., 2011; Kärst et al., 2011; Grilz-Seger et al., 2019).

We observed selection at the loci of other top-ranking candidates from the haplotype analysis with known roles in energy consumption, liver metabolism, milking, fat deposition, and angiogenesis (Yang et al., 2016; Armstrong et al., 2018; Pszczola et al., 2018). These findings, showing the top-ranking genes sharing functions of that in the resequencing study, provide many candidate genes relating to survivability and socioeconomic traits in Welsh upland sheep. Further evidence of selection in these breeds can be seen from functional term enrichments in genes found in the selected regions and in CNVRs.

Lowland breeds showed less signatures of selection, however, they mainly had selection in regions containing genes known to be associated with domestication, which are commonly reported signatures of selection in world sheep breeds (Kijas et al., 2012; Wei et al., 2015; Wang et al., 2019; Li et al., 2020). By demonstrating the lowland breeds sharing the signatures of selection with other productive breeds, this indicates that they are better suited for productive qualities but do not show an adaptation for the Welsh uplands. Despite this, selection for *CDK5RAP2* seen in the lowland breeds of the resequencing study may be linked to upland adaptation as mice models with truncating mutations have lower red blood cell counts, however, this is true for white blood cells too, and so, may be linked to immunity traits as well as neurodevelopment (Barker and Bernstein, 1983).

Differences in the results when using the genotyping and resequencing data were expected and can be attested to an increased density of SNPs in the resequencing data and a different composition of populations used for each study. The former effect would be expected to lead to narrower regions being detected under selection in the resequencing dataset, which could shift the most significant SNP away from candidate genes detected from the genotyping data. Secondly, using groups of multiple breeds could mean that whilst haplotypes often remain similar amongst closely related breeds, certain point mutations that differentiate those breeds from each other may become diluted in frequency, and so, would not be considered under selection by this method when a small number of animals is used. This is likely the case where, lowland breeds show less signatures of selection than upland breeds, suggesting that they share less signatures when grouped. This postulates that Welsh lowland breeds are more diverse than the upland breeds, supported by data from Beynon et al. (2015), which could be in response to the demand for socioeconomic traits and lack of selection pressures in comparison to the upland breeds, where there seem to be a selective pressure on the same region, leading to shared signatures of selection. This further suggests that the lowland breeds have been selected for the production of socioeconomic traits, rather than adaptation to their local environment.

Lower costs of genome resequencing have allowed a deeper insight to the individual mutations that could have functional roles within a region under selection; however, this is not always a realistic approach when investigating many related breeds in a single study. Our method here has demonstrated reliability in using resequencing data from a small number of individuals of different breeds but applied to similar environments can be supported by genotyping many individuals of these breeds. This is truer with the upland Welsh breeds, which is most likely due to the higher environmental selective pressures applied when compared to the lowland breeds. This has greatly eluted candidate genes relating to hardiness and survivability in both the genotyping and resequencing data.

## Conclusion

Here, we have seen the first investigation into signatures of selection in Welsh sheep breeds using a large number of genotyped individuals and a small number of whole-genome resequenced individuals. Statistical pipelines have shown selection in Welsh upland breeds in regions containing genes relating to adaptation to the local environment, including candidate genetic variants, as well as some genes related to the production of socioeconomic traits in the lowland breeds. In turn, this information is useful, not only for the conservation of these culturally important breeds, but also for the improved production capabilities of mountain breeds and adaptation of productive breeds through a marker-assisted selection.

## Data Availability Statement

The original contributions presented in the study are publicly available in NCBI using accession number PRJNA646642.

## Author Contributions

DL: leading the project, sample collection, and writing the manuscript. JS-J: running the analyses and drafting the manuscript. VL: genome sequencing of Welsh sheep samples and initial analysis. AY: analysis pipeline development. NY: sample collection and manuscript editing. MS: Welsh sheep sequencing and initial analysis. All authors edited the manuscript.

## Conflict of Interest

The authors declare that the research was conducted in the absence of any commercial or financial relationships that could be construed as a potential conflict of interest.

## Footnotes

1 http://broadinstitute.github.io/picard/
