# Supplementary material for: Genotyping and Whole-Genome Resequencing of Welsh Sheep Breeds Reveal Candidate Genes and Variants for Adaptation to Local Environment and Socioeconomic Traits
Source: Front Genet. 2021 Jun 18;12:612492. doi: 10.3389/fgene.2021.612492 (PMC8253514; doi:10.3389/fgene.2021.612492)
Supplement: Supplementary file 1 [file Data_Sheet_1.PDF]

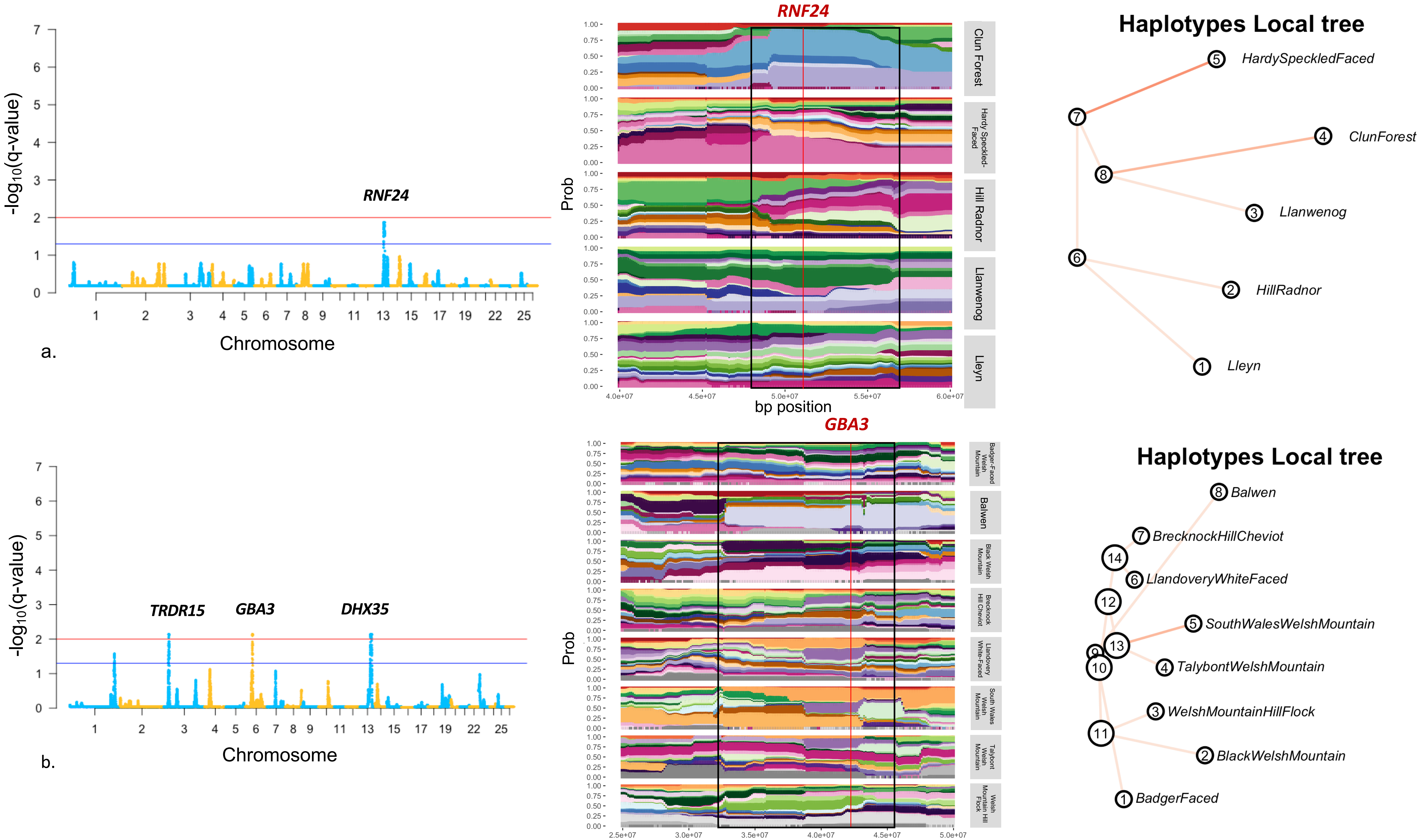

-log10(q-value)
RNF24
Chromosome
a.
RNF24
Prob
bp position
Clun Forest
Hardy Speckled-Faced
Hill Radnor
Llanwenog
Lleyn
Haplotypes Local tree
HardySpeckledFaced
ClunForest
Llanwenog
HillRadnor
Lleyn
-log10(q-value)
TRDR15
GBA3
DHX35
Chromosome
b.
GBA3
Prob
bp position
Balwen
Haplotypes Local tree
Balwen
BrecknockHillCheviot
LlandoveryWhiteFaced
SouthWalesWelshMountain
TalybontWelshMountain
WelshMountainHillFlock
BlackWelshMountain
BadgerFaced

Figure S1

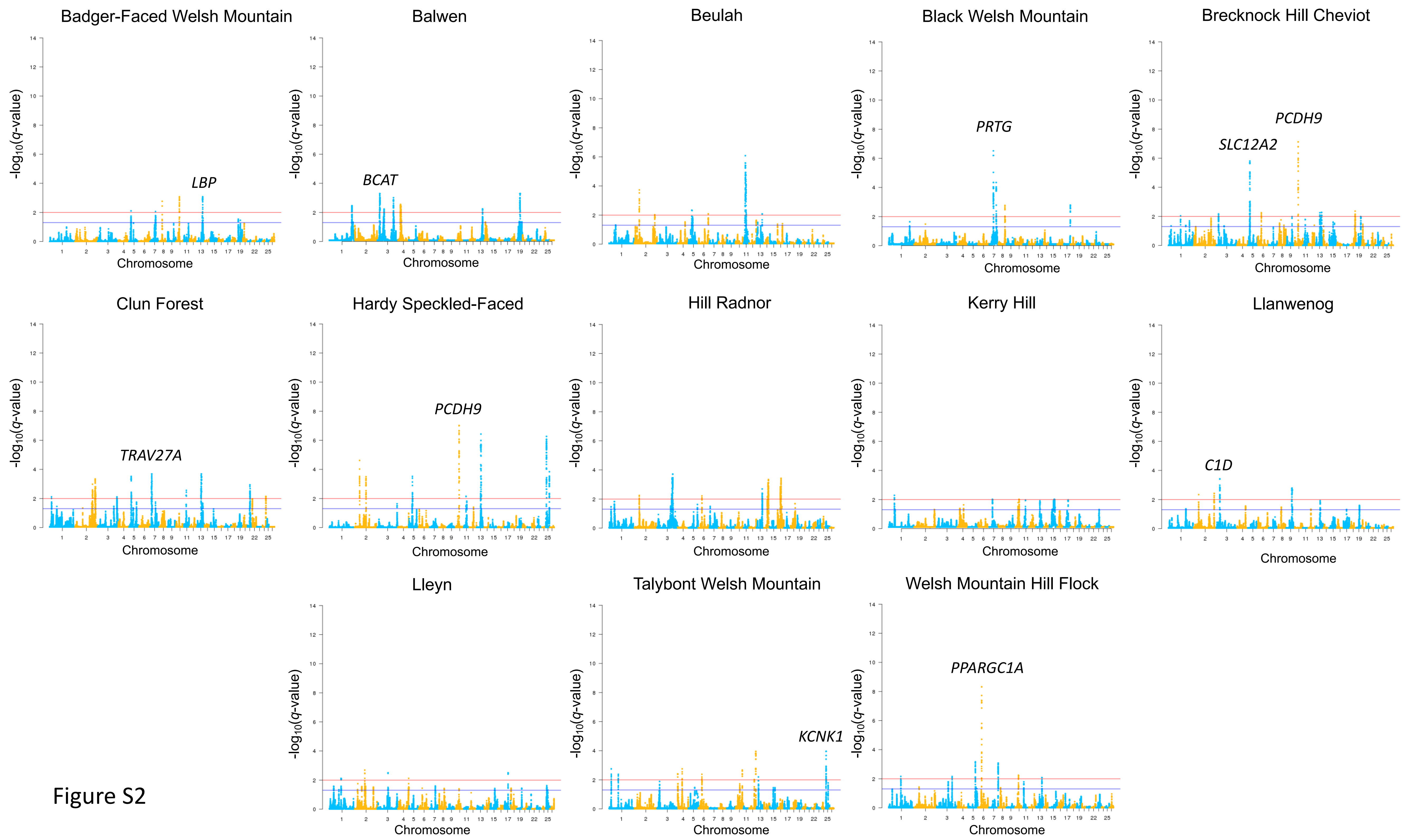
Badger-Faced Welsh Mountain
LBP
Balwen
BCAT
Beulah
Black Welsh Mountain
PRTG
Brecknock Hill Cheviot
SLC12A2
PCDH9
Clun Forest
TRAV27A
Hardy Speckled-Faced
PCDH9
Hill Radnor
Kerry Hill
Llanwenog
C1D
Lleyn
Talybont Welsh Mountain
KCNK1
Welsh Mountain Hill Flock
PPARGC1A
-log10(q-value)
Chromosome

Figure S2

Figure S1: Output of most strongly selected regions of Lowland (a) Upland (b) of HapFLK study. This includes Manhattan plot with gene proximal to most significant SNP of highly selected peaks. Cluster plot show tracks of haplotype diversity for the peak highlighted on Manhattan plot shown by black border where the red line represents the most significant SNP. Finally a Haplotype Local Tree produced by HapFLK shows strong selection for each region by individual breeds through increasing intensity of branch colour.

Figure S2: Manhattan plots of all genotyped breeds' DCMS output. Selection thresholds for suggestive (q-value < 0.05) and strong (q-value < 0.01) selection shown by blue and red lines respectively. Significant selection peaks have been annotated with the name of the top-ranked gene of that region.
